# Physicians’ quality of life, illness and presenteeism: a cross-sectional epidemiological study

**DOI:** 10.47626/1679-4435-2022-743

**Published:** 2023-02-03

**Authors:** Jonas Munck de Oliveira, Laryssa de Sá Bragança Gonçalves, Ana Luísa Scafura da Fonseca, Lívia Ferreira dos Santos, Matheus Bresser, José Antonio Chehuen-Neto, Renato Erothildes Ferreira

**Affiliations:** 1 Núcleo de Cirurgia Experimental, Faculdade de Medicina, Universidade Federal de Juiz de Fora, Juiz de Fora, MG, Brazil.; 2 Faculdade de Medicina, Universidade Federal de Juiz de Fora, Juiz de Fora, MG, Brazil.; 3 Departamento de Cirurgia, Faculdade de Medicina, Universidade Federal de Juiz de Fora, Juiz de Fora, MG, Brazil.; 4 Pós-Graduação em Saúde, Faculdade de Medicina, Universidade Federal de Juiz de Fora, Juiz de Fora, MG, Brazil.

**Keywords:** physicians, quality of life, occupational health, presenteeism, medical assistance, médicos, qualidade de vida, saúde do trabalhador, presenteísmo, assistência médica

## Abstract

**Introduction:**

The World Health Organization defines quality of life as “ an individuals’ perception of their position in life, in the context of the culture and value systems in which they live and in relation to their goals, expectations, standards, and concerns.” physicians, when dealing with illness and exposing themselves to the risks of their profession, must act without compromising their own health status in view of the function performed.

**Objectives:**

To evaluate and correlate physicians’ quality of life, professional illness, and presenteeism.

**Methods:**

This is an epidemiological, cross-sectional, descriptive study with an exploratory quantitative approach. Overall, 309 physicians working in Juiz de Fora, state of Minas Gerais, Brazil were interviewed and answered a questionnaire with sociodemographic and health information and the World Health Organization Quality of Life instrument-Abbreviated version (WHOQOL-BREF).

**Results:**

Of physicians in the sample, 57.6% fell ill during their professional activities, 35% took sickness absence, and 82.8% practiced presenteeism. The most prevalent diseases were those involving the respiratory system (29.5%), infectious or parasitic diseases (14.38%), and those involving the circulatory system (9.59%). WHOQOL-BREF scores were boas, and were influenced by sociodemographic characteristics such as sex, age, and time of professional experience. Male sex, professional experience greater than 10 years, and age above 39 years were associated with beter quality of life. Previous illness and presenteeism were negative factors.

**Conclusions:**

The participating physicians had a good quality of life in all domains. Sex, age, and time of professional experience were relevant factors. The highest score was observed in the physical health domain, followed by psychological domain, social relationships, and environment, in a descending order.

## Abstract (pt)

**Introdução:**

A Organização Mundial da Saúde define qualidade de vida como “a percepção do indivíduo de sua inserção na vida, no contexto da cultura e nos sistemas de valores nos quais ele vive e em relação a seus objetivos, expectativas, padrões e preocupações”. Os médicos, ao lidarem com pacientes e se exporem aos riscos da profissão, devem atuar sem comprometer seu estado de saúde frente à função desempenhada.

**Objetivos:**

Avaliar e correlacionar a qualidade de vida, o adoecimento profissional e o presenteísmo do médico.

**Métodos:**

Estudo epidemiológico de corte transversal, descritivo e exploratório, com características quantitativas. Foram entrevistados 309 médicos atuantes em Juiz de Fora, no estado de Minas Gerais, submetidos a questionário com informações sociodemográficas e de condições de saúde e ao World Health Organization Quality of Life instrument-Abbreviated version (WHOQOL-BREF).

**Resultados:**

Na amostra, 57,6% adoeceram durante a atuação profissional, 35% se afastaram do trabalho e 82,8% praticaram presenteísmo. As doenças mais prevalentes foram do sistema respiratório (29,5%), infectoparasitárias (14,38%) e do sistema circulatório (9,59%). As pontuações no WHOQOL-BREF foram boas, afetadas por características sociodemográficas como sexo, idade e tempo de atuação profissional. Sexo masculino, atuação superior a 10 anos e idade maior que 39 anos foram associados a melhor qualidade de vida. Adoecimento prévio e presenteísmo foram fatores negativos.

**Conclusões:**

Os médicos participantes do estudo apresentaram boa qualidade de vida em todos os domínios. Sexo, idade e tempo de atuação profissional mostraram-se fatores relevantes. A melhor nota foi observada no domínio físico, com pontuações decrescentes nos domínios psicológico, social e ambiental.

## INTRODUCTION

The expression “quality of life” was coined by the then American president Lyndon Johnson, in the 1960s, when stating that the “goals cannot be measured by the balance sheet of banks. They can only be measured by the quality of life they provide to people.” Regardless of the sociocultural context, each individual should feel psychologically well, in a good physical condition, socially integrated, and functionally competent.^1^

The World Health Organization (WHO) defines quality of life as “an individuals’ perception of their position in life, in the context of the culture and value systems in which they live and in relation to their goals, expectations, standards, and concerns.”^1^ From this perspective, it is important that physicians, when dealing with illness and exposing themselves to the risks inherent to professional practice, must act without compromising their own health status in view of the function performed.^2^

The discussions of the VIII Brazilian National Health Conference and of the I Brazilian National Occupational Health Conference, in line with the Brazilian democratic transition and with what was occurring in the Western world, provided a new decisive focus for the theme, with the changes established in the new 1988 Federal Constitution.^3^ Updated to multicausality theory and developed interprofessionally, occupational health adopts the perspective that a set of risk factors is considered in the production of disease, being assessed from the perspective of medical clinical and by environmental and biological indicators of exposure and effect.^4^

Occupational risks are divided into five groups, according to their nature: physical risks include exposure to noise, extreme temperatures, and ionizing radiation; chemical risks are represented by gases, vapors, and various reagents; biological risks cover exposure to micro-organisms, blood, and blood-derived products; ergonomic risks refer to inadequate posture, repetitiveness, work in shifs, and stressful situations; and risks of accident encompass inadequate arrangement of workplace, insuficient lighting, potential accidents with electricity, and probability of fre.^5^

Different risk groups may be present in medical activity, in addition to the specific demands related to the work of these professionals. The literature shows that, compared with other professionals, physicians’ vacation time is nearly half shorter and their average weekly working hours is 15 hours longer.^6^ The observed prevalence of presenteeism, i.e., atending work while sick, is 80 to 90% among medical professionals, in comparison with 30 to 70% in other professions.^7^ Due to ergonomic issues of their activity, 59% of surgeons present with neck pain, observed in 20% of the general population.^8^ Dissatisfaction with work-life balance is also higher than that of population controls, 40.2% *vs.* 23.2%, respectively.^9^ In a research conducted by the Medical Society of the State of New York with more than 1,000 physicians, 57% of respondents had symptoms of burnout.^10^

According to the WHO Declaration on Workers’ Health, there is a growing recognition about the linkages between working conditions, health, and productivity.^11^ Therefore, physicians’ perception about their own quality of life collaborates closely to investigate the interdependence between their extenuating professional activity and the several aspects of their life, even afecting their ability to provide quality care to patients who seek them.^12^ It is known that 74% of diagnostic errors are related to cognitive aspects of these professionals, depending not only on good training and level of knowledge, but also on good reasoning and interpretation.^12^ These cognitive aspects are compromised by burnout, which is a very common condition among professionals who sacrifice their well-being in favor of profession activity and are subject to various occupational risks.^6^

This research collected data up to the beginning of the coronavirus disease 2019 (COVID-19) pandemic, a disease caused by the severe acute respiratory syndrome coronavirus 2 (SARS-CoV-2), which emerged in December 2019 in Wuhan, China. On March 11^th^, 2020, the WHO declared COVID-19 as a pandemic, thus ratifying and exacerbating the frequent physical, ergonomic, and psychological risks of health care professionals. Increased workload, physical exhaustion, inadequate personal equipment, nosocomial transmission, and need of making ethically difficult decisions on rationing of care may have dramatic effects on physical and mental well-being. This highlights the urgent need for beter interventions on health care providers’ physical and mental health.^13^

The present study aimed to assess physician’s quality of life and aspects that afect it, as well as professional illness and presenteeism, in addition to correlating these factors with physician’s quality of life.

## METHODS

An epidemiological, cross-sectional, descriptive study with an exploratory quantitative approach was performed. It consisted of an original survey conducted with physicians in the city of Juiz de Fora, state of Minas Gerais, Brazil. As a standardization criterion, the area of study was delimited as the geographic area with the greatest concentration of doctor’s ofices and hospitals. Therefore, the study was conducted in the city’s downtown, because care provision is centralized in this area, thus characterizing a plural and representative sample for this study.

According to a survey performed by the Brazilian Federal Council of Medicine (Conselho Federal de Medicina, CFM), Juiz de Fora had 3,191 physicians in 2018, working and living in the municipality. Of these, 2,405 were registered as specialists.^14^

In a literature review, it was not possible to obtain an accurate and reliable prevalence of illness in the medical population; therefore, to estimate the sample, the hypothetic prevalence of illness leading to sick leave was set at least 20% per year.^15^ According to Levine et al.,^15^ when p and q values are unknown, the following equation should be used to calculate the sample: $n=\left({\left(Z_{\frac{a}{2}}\right)}^{2}\times \text{hypotethical p-value}\right)/E^{2}$ . This equation is used to compare proportions with categorical data in an independent sample in which population parameters are unknown, for a 95% confdence interval (95%CI) and alpha of 0.05. This ensures representativeness for the sample of physicians from the municipality of Juiz de Fora with a maximum error of ± 5%.^15^ For estimates with no continuity correction, the sample was estimated at 309 individuals.

Inclusion criteria were being a physician working in Juiz de Fora and agreeing to participate in the research by signing the informed consent form (ICF). Incomplete questionnaires were considered sample loss. Exclusion criteria consisted of physicians who were not practicing their profession.

Information was obtained using a structured questionnaire containing 40 questions divided into two parts. The first part consisted of 14 questions developed by the researchers and that enabled to characterize the sample with regard to sociodemographic aspects, alcohol consumption, and smoking, in addition to previous illnesses and absence from work activities. In the second part, quality of life was assessed using the World Health Organization Quality of Life instrument-Abbreviated version (WHOQOL-BREF) questionnaire, which contained 26 questions, two general questions on quality of life and the others related to the following domains: physical health, psychological, social relationships, and environment. This instrument does not provide an overall quality of life score, because it assumes the premise that quality of life is a multidimensional construct; thus, each domain is score independently. The final score is transformed into a 100-point scale, in which 0 equals the worst quality of life and 100, the beter.

Data collection was conducted from July 2019 to February 2020 by five duly trained students from the School of Medicine of Universidade Federal de Juiz de Fora, so that the questionnaire items were applied and explained in a standardized way. Researchers’ training occurred in a pilot study conducted with 20 volunteer physicians in order to identify problems in the understanding of instruments and to define approach and approximation criteria, thus ensuring the quality of data collection. Researchers made previous contact with hospitals, clinics, and professionals via institutional email to schedule the interview. Volunteers were approached in their workplaces and during their work hours. Afer in-person presentation, explanation on research goals, and clarifications on the confdentiality of information and data contained in the forms, professionals were invited to answer printed questionnaires. Researchers did not insist in case of refusal, even afer previous contact. Participants could withdraw from the research at any time if they so requested. Refusal rates were low, being estimated at one for every five physicians approached, and were most related to lack of time availability from the professional. No participant withdrew or was excluded from the study.

Data were double-typed and double-checked so as to mitigate possible inconsistencies or typos.

Data were described by measures of central trend and dispersion, as appropriate depending on level of measurement and symmetry of distribution for the variable of interest, i.e., means, standard deviations, medians, range, confdence intervals, and absolute (n) and relative (%) values.

Odds are frequently assessed using a measure of association or a measure of effect, which translates the association between exposure and outcome. Teoretically, these indicators measure the strength of the association between epidemiological variables. In this study, with respect to analysis of data with binary outcomes, the measure of association called odds ratio (OR) was used, especially because this measure (estimator) express risk. OR assesses the relationship between the odds of individuals exposed to have the condition of interest for the study, compared to that of non-exposed ones.^16^

The effects of the association between exposure and outcome of “quality of life, illness, and medical presenteeism” (QLIMP) was measured using a multivariate logistic regression model to estimate OR. The multivariate model followed a forward-driven approach, i.e., a method to select variables grouped into dimensions considering the proximity of each dimension with a potential modifer of the QLIMP outcome. The final model considered all variable with effect >25% in modules (increased or reduced odds), p-value < 0.20, and 95%CI that did not contain (1).

The selection for the final multivariate model that beter explained the study outcome was defined by two criteria. Firstly, by pseudo-R^2^; in contrast to minimum ordinary R^2^-squared, pseudo-R^2^ is based on a likelihood logarithm and did not represent the proportion of the explained variance, but rather the increased probability of the model to contain explanatory variables in relation to a null hypothesis model, which only contains the constant.^16,17,18^ Secondly, by the final quality of adjustment based on the Akaike’s information criteria (AIC).^16,17,18^ AIC admit the existence of a “true” model which describes data that are unknown in the study and tries to choose between a group of models assessed, which minimizes the Kullback–Leibler divergence related to information that is naturally lost when using an approximate model and not the “true” one, i.e., the one that contained the true parameter in the population.^16,17,18^ The model with beter adjustment was the one that presented lower AIC values.

The level of significance was alpha ≤ 0.05 for a 95%CI. Analyses were conducted using Stata 15 sofware (Data Analysis and Statistical Sofware College Station, Texas, USA).

The research was approved by the Research Ethics Commitee of Universidade Federal de Juiz de Fora under opinion n. 2.642.932 and Certificate of Presentation for Ethical Appraisal (Certificado de Apresentação de Apreciação Ética) 87463018.8.0000.5147. Reliability and privacy criteria were ensured to participants according to Resolution n. 466/2012 of the Brazilian National Health Council, which addresses research involving human beings.

## RESULTS

The study assessed 309 physicians of 38 specialties working in the city of Juiz de Fora, state of Minas Gerais, Brazil. The sample consisted mainly of women, 54.4% (n = 168), and mean age was 40.9 ± 12.8 years, ranging from 24 to 79 years, and median of 39 years. Mean time of profession for the group was 14.3 ± 12.8 years, ranging from 1 to 50 years, and with median of 10 years, which suggests that the group had a significant number of young physicians at the initial stage of their career.

More than a half of participants worked in the large area of clinical medicine (53.1%, n = 164), one fourth of them worked in surgical specialties (25.2%, n = 78); 11.3% (n = 35), in gynecology and obstetrics; 9.4% (n = 29), in pediatrics; and only 1% (n = 3) worked in family practice.

Of the research participants, 57.6% (n = 178) reported falling ill during their professional activities, totaling 292 episodes of illness and 109 different diseases, which were divided according to the 24 chapters of the International Classification of Diseases and Related Health Problems, eleventh revision (ICD-11)^19^ (Table 1).

**Table 1:** Distribution of burden of diseases reported by physicians according to the chapters of the International Classification of Diseases and Related Health Problems (ICD-11)^19^

ICD-11 chapter	n	%
Certain infectious or parasitic diseases	42	14.38
Neoplasms	4	1.36
Diseases of the blood or blood-forming organs	0	0.00
Diseases of the immune system	2	0.68
Endocrine, nutritional, or metabolic diseases	9	3.08
Mental, behavioral, or neurodevelopmental disorders	18	6.17
Sleep-wake disorders	1	0.34
Diseases of the nervous system	12	4.11
Diseases of the visual system	7	2.40
Diseases of the ear or mastoid process	2	0.68
Diseases of the circulatory system	28	9.59
Diseases of the respiratory system	86	29.50
Diseases of the digestive system	11	3.76
Diseases of the skin	0	0.00
Diseases of the musculoskeletal system or connective tissue	11	3.76
Diseases of the genitourinary system	11	3.76
Conditions related to sexual health	0	0.00
Pregnancy, childbirth, or the puerperium	2	0.68
Certain conditions originating in the perinatal period	0	0.00
Developmental anomalies	0	0.00
Symptoms, signs, or clinical findings, not elsewhere classified	10	3.42
Injury, poisoning or certain other consequences of external causes	20	6.85
External causes of morbidity or mortality	9	3.08
Factors influencing health status or contact with health services	7	2.40

Among respondents, 82.8% (n = 256) reported having already practiced presenteeism. Absence from work was reported by 35% (n = 110); of these, 64.5% were absent once; 22.7%, twice; 5.5%, three times; 5.5%, from four to nine times; and 1.8%, 10 times or more. Mean number of days absent from work was 23 ± 83 days, ranging from 1 to 730 days. Among participants, 52.1% (n = 161) have sick leave insurance, and 80.9% (n = 250) do not feel represented by entities representing their professional category with regard to quality and safety of their work.

Consumption of alcohol was reported by 61.8% (n = 191); of these, 7.3% consumed alcoholic beverages less than once a week; 50.3%, once a week; 24.6%, twice a week; 12%, three times a week; and 5.8%, four times a week or more. Smoking was reported by 4.5% (n = 14), of which 57% smoked less than a packet of cigaretes per week.

Answers to the WHOQOL-BREF questionnaire were divided into its respective domains and transformed into scales ranging from 0 to 100 points (Table 2).

**Table 2:** Summary of descriptive statistics (measures of central tendency and dispersion) most used in publications in the health area related to generic questions on quality of life and domains of the World Health Organization Quality of Life instrument-Abbreviated version (WHOQOL-BREF)

Generic questions on quality of life and WHOQOL-BREF domains	Mean ± SD	Median	Amplitude
How would you rate your quality of life?	72.5 ± 18.6	80.0	20-100
How satisfied are you with your health?	73.2 ± 18.5	80.0	20-100
Physical health	75.3 ± 13.8	75.0	35.7-100
Psychological domain	70.2 ± 14.5	70.8	25-100
Social relationships	70.3 ± 14.9	75.0	25-100
Environment	67.7 ± 21.9	68.8	21.9-100

SD = standard deviation.

Subsequently, a multivariate regression analysis was performed associating sociodemographic aspects, alcohol consumption, smoking, previous illnesses, and sickness absence from work with domain scores (Table 3).

**Table 3:** Multivariate analysis showing adjusted odds ratio (OR) for independent variables, followed by probabilities (p-value), 95% confidence intervals (95%CI), and measures of quality of model adjustment (pseudo-R^2^ and Akaike’s information criteria [AIC]) for the six dimensions studied

Variable	OR	p-value	95%CI	Pseudo-R^2^	AIC
MODEL 1: How would you rate your quality of life?					
Male sex	1.16	0.635	0.5-1.99	22.09	326.4
Time of professional experience ≥ 10 years	2.05	0.013	1.16-3.64		
Physical health (≥75^th^ percentile)	3.48	0.001	1.88-6.41		
Psychological domain (≥75^th^ percentile)	3.62	0.003	1.55-8.42		
Environment (≥75^th^ percentile)	2.14	0.078	0.86-5.27		
MODEL 2: How satisfied are you with your health?					
Male sex	1.72	0.065	0.96-3.06	19.37	334.4
Time of profession ≥ 10 years	1.97	0.022	1.10-3.54		
History of illness	2.1 6	0.012	1.18-3.91		
Practices presenteeism	2.48	0.047	1.01-6.08		
Physical health (≥75^th^ percentile)	3.95	0.001	2.25-7.29		
Psychological domain (≥ 75^th^ percentile)	3.41	0.001	1.60-7.29		
MODEL 3: Physical health					
Male sex	1.62	0.046	1.00-2.58	5.00	415.2
Age ≥ 39 years	2.00	0.004	1.24-3.23		
History of illness	1.91	0.008	1.18-3.08		
MODEL 4: Psychological domain					
Male sex	1.26	0.375	0.75-2.12	3.40	348.7
Age ≥ 39 years	0.47	0.113	0.19-1.19		
Time of professional experience ≥ 10 years	2.04	0.001	1.35-3.08		
History of illness	1.30	0.327	0.77-2.19		
Does not practice presenteeism	1.70	0.105	0.89-3.24		
MODEL 5: Social relationships					
Male sex	3.04	0.001	1.66-5.54	5.80	332.4
Age ≥ 39 years	0.56	0.264	0.21-1.53		
Time of professional experience ≥ 10 years	1.40	0.123	0.91-2.16		
History of illness	1.62	0.082	0.92-2.85		
Practices presenteeism	1.53	0.263	0.72-3.33		
MODEL 6: Environment					
Male sex	1.81	0.040	1.02-3.20	19.90	345.4
Age ≥ 39 years	0.21	0.002	0.07-0.57		
Time of professional experience ≥ 10 years	2.28	0.001	1.46-3.56		

Model 1 shows the best values in adjustment criteria, lower AIC value (326.4), and higher pseudo-R^2^ value (22.09). This means that the variables in this model explain 22.09% of variance in the scores for the question “how would you rate your quality of life?”, and the independent variables sex (OR 1.72; 95%CI 0.96-3.06; p-value 0.065), time of profession ≥ 10 years (OR 2.05; 95%CI 1.16-3.63; p-value 0.013), physical health (OR 3.48; 95%CI 1.88-6.41; p-value 0.001), psychological domain (OR 3.62; 95%CI 1.55-8.42; p-value 0.003), and environment (OR 2.14; 95%CI 0.86-5.27; p-value 0.078) were associated with greater odds of increased scores in this question.

Model 6 shows the second highest pseudo-R^2^ value (19.8), i.e., it explains nearly 20% of variance in scores for the environment domain, exerting a great effect on the variables sex (OR 1.81; 95%CI 1.02-3.20; p-value 0.040), age ≥ 39 years (OR 0.21; 95%CI 0.07-0.57; p-value 0.002), and time of profession ≥ 10 years (OR 2.28; 95%CI 1.46-3.56; p-value 0.001). Age had a protective effect; therefore, physician older than 39 years have nearly 79% lower odds of obtaining a poor score in the environment domain. In model 4, age also exerted a protect effect and led to 53% lower odds of low scores in the psychological domain, but with no statistical significance (p-value 0.113).

## DISCUSSION

Studies addressing quality of life in Brazil are relatively recent and have increased every year. Most of them are not limited to a certain social or professional group, but rather to patients with some disease.^20^ Our literature review found few texts that enabled to establish a scientific comparison, because there is a scarcity in investigations addressing overall quality of life among the medical population. The dimension of Brazil and its remarkable regional characteristics hamper this comparison, considering the influence of culture on individual’s perception of quality of life.

The COVID-19 pandemic that ravaged the country exacerbates a characteristic presented by our research: although the risks of medical practice involving quality of life are evident, they are also ofen hidden in the precarious working conditions in general, which are overcome by professionals with professional dedication and diligence. A systematic review and meta-analysis assessing 13 studies with more than 30 thousand physicians found a pooled prevalence of 23.2% for anxiety and 22.8% for depression.^13^ Previous studies about SARS and Ebola epidemics indicated that the onset of a sudden and immediate life-threatening illness could lead to an extraordinary amounts of pressure on health care professionals, resulting in high rates of depression and anxiety, as well as post-traumatic stress disorder.^13^

Physicians have experienced even more significant physical, ergonomic, and psychological risks during the COVID-19 pandemic. Strenuous workload, inappropriate personal protective equipment, and the risk of nosocomial transmission, among others, have catastrophic effects on physicians’ physical and mental state. Teir resilience may be even more compromised by isolation and loss of social and risk or infections of friends and relatives, as well as drastic, ofen unsetling changes in the ways of working.^13^ The number of confirmed cases of COVID-19 among health care professionals in general, according to the Brazilian Ministry of Health, 2021, exceeded 442 thousand up to January 2^nd^, including 48,859 physicians,^21^ of which 551 died up to December 2020, according to the CFM.^22^

Physicians interviewed in Juiz de Fora reported high quality of life, based on the assessment model proposed at the time of data collection. This is a medium-sized city; thus, distances to commute to work are shorter, trafic has known botlenecks that can be avoided, and levels of criminality are lower than those of the large Brazilian cities, factors that also contribute to a good quality of life for the general population. The quality of education and health services is high in all levels, compared to most Brazilian municipalities.

Quality of life indicators for participating physicians exhibited higher scores than those observed in the four domains included in a study with 1,110 out of the 6,100 physicians registered at the Regional Medical Council of the state of Paraíba, Brazil.^23^ Score for male respondents were higher than those of females, which was also observed in the study conducted in Paraíba.^23^ Considering a 100-point scale, scores were relatively good, ranging within the threshold of the third quartile, as well as the assessment of individuals’ own quality of life and health.

The worst score was observed in the environment domain, a result that is probably related to the particularities of health organization in the city. Additionally, the interviewed physicians reported great dissatisfaction in relation to the entities that represent their professional category, in terms of occupational quality and safety, since 80.9% of workers did not feel represented by these entities.

According to the analysis of the data obtained, being a male, having more than 10 years of professional experience, and obtaining a high score in the physical health and psychological domains led to greater odds of individuals rating their own professional quality of life as good and of having greater satisfaction with their own health. In an American study, physicians who had already completed residency showed higher rates in the following domains: physical health, psychological, and environment.^24^

Good scores in the psychological domain are related to male sex, previous illness, no history of presenteeism, and time of profession of 10 years or more. These numbers indicate the positive influence of greater maturity for a beter psychological well-being. With regard to disparity between sexes in this domain, it may be related to the fact that women are more concerned about time for their relationships than men,^25^ whereas many women also have to balance their professional, maternal, and marital roles.^6^

The environment domain had the lowest mean and the lowest minimum score, being positively influenced by male sex and time of profession of 10 year or more. This is an extremely relevant aspect, because 84% of recently graduated physicians consider working conditions as relevant, and 45.7% value safer environments.^26^ A European study observed that female physicians were more likely to be dissatisfed with their safety and living places than their male counterparts, with a relative risk of 2.51,^27^ a situation corroborated by our study.

An American systematic review pointed that men physicians had higher scores in the social relationships domain than women physicians, being more satisfed with friends, family, colleagues, and bosses, as well as with career-advancement opportunities, recognition, and salary.^25^ This scenario is corroborated by our study, since the social relationships domain was more positive in the male sex.

Some factors inherent to the female universe, such as pregnancy, delivery, breastfeeding, and involvement in children’s education, in addition to the medical work itself, may be associated with lower scores in the following domains: psychological, physical health and environment domains, since women experience double burden of work in their personal and professional life, whereas some factors, such as leisure and availability, are less present in the life of female professionals.^23^

In relation to illness during work activities, most participants reported that they had already fell ill (57.6%), whereas 35.6% reported taking a sick absence from work, an absence that lasted for 23 days, on average, and was repeated for three times or more among 12.8% of physicians. These numbers would be even greater if there was no trivialization of less severe conditions in the group of relatively young professionals and if presenteeism was not so common among physicians. This fact was corroborated in our research, since 82.8% of respondents stated having atended work while feeling sick, although a smaller portion had reported falling ill during medical practice.

Presenteeism is a phenomenon observed in several countries, showing various rates and causes. In National Health Service (NHS), the United Kingdom’s health system, sickness absence rates among physicians were less than a third of rates across the system as a whole, and they were absent because of sickness leave on only 1.3% of the days they were scheduled to work, in comparison to an average sickness absence rate of 4.5% for nurses and 5.5% for ambulance workers.^28^ In New Zealand, 88% of the 1,806 physicians participating in a study reported that they had already atended work while feeling sick to maintain their usual standards of performance.^29^ Among 107 Israeli physicians, only 17.8% said that they did not come to work ill,^30^ a percentage similar to the 17.2% of participants who reported doing so in our research.

In addition to organizational, cultural and social components that motivate presenteeism, there are others whose correlation is more evident, such as appreciation and awards to longer working hours and to high workload, inability of others to take over duties, negative sanctions from colleagues or management, and feelings of moral obligation.^31^ However, this atitude may be motivated by inability to remain financially stable during sickness absence,^31^ a situation that could apply to the respondents of this study, since slightly more than a half of participants (52.1%) are covered by labor insurance services.

In our study, the most prevalent diseases during medical practice were those involving the respiratory system (29.50%), infectious or parasitic diseases (14.38%), and those involving the circulatory system (9.59%). These findings are somewhat different from the causes of sickness absence among NHS physicians, with a higher prevalence of musculoskeletal problems, present in 18.1% of the cases, and stress and depression in 7.3%, whereas no evidence of infectious or parasitic diseases was observed in this group.^28^ Although our sample had a different nosologic profile, it was found that the percentage of mental disorders is close to that of the British study, accounting for 6.17% of diseases,^28^ whereas diseases of the musculoskeletal disorders accounted for 3.76%. This similarity in the percentage of mental disorders show that, despite ethnical, cultural, and epidemiological diferences, physicians from Juiz de Fora have a burden of stressors similar to that of physicians from the United Kingdom. These stressors associated with the profession may be related to medical practice itself, such as few hours of sleep and fear of medical error, or related to individual’s personality in relation to medicine, such as patients’ idealization of physicians, constant and intense contact with pain, death, and sufering, working with emotional and physical intimacy, and relationship with difficult patients.^6^

The professional activity of Brazilian physicians is characterized by extenuating working shifs, great variety of atributions, low payment, and occupational burnout, a consensual view for more than a half of these professionals,^32^ with 57% displaying a worrying level of burnout.^33^ However, our study found that only 2.4% of participants sufered from diseases related to factors influencing health status or contact with health services, including burnout. This diference is possibly explained by the fact that we did assess symptoms of disease itself; instead, we collected a general report from professionals about conditions that had already been diagnosed, which may suggest that this disease is underdiagnosed among our respondents.

Suicidal rates are a serious issue among physicians, being higher than those of the general population,^34^ which may evidence a possible neglect of this professional category. Furthermore, long working hours and the typical features of physicians’ personality^6^ may contribute to the development of depression and anxiety, with need for psychological support. Our study found that 10.1% of the physicians who had already fell sick during their professional activities present or had already presented some mental or behavioral disorder, which accounts for 6.17% of diseases, including depression, anxiety, and panic disorder, diseases that are closely related to suicide.

With regard to alcohol consumption, a German study reported that 82.5% of physicians consumed alcohol at least once a week, and 50.1% reported higher ingestions than the recommended daily allowance by the German Nutrition Society – up to 20 grams/day for men and 10 grams/day for women.^35^ Our study found that 61.8% of professionals drank alcohol, and 11% drank it three times a week or more. However, it is worth considering that there is no safe limit for the consumption of alcohol, according to the WHO.^36^ High consumption of alcohol is associated with 60 different conditions, such as cancer and increased blood pressure, usually having a high correlation with the ingested amount.^36^ This habit is ofen acquired during early adulthood, during the training period of these professionals, when intense course load, competitiveness among colleagues, sleep deprivation, fear of making a mistake, and others stressors are compensated for by the use of illicit and licit drugs, such as alcohol.^37^ In a medical school in the state of Minas Gerais, Brazil, it was observed that 83.5% of students consumed alcohol, of which 39.6% could be classified as heavy users, and 2.8% as being in a situation of alcohol dependence syndrome.^37^

As for smoking, it was reported by only 4.5% of the physicians interviewed, and 8 out of the 14 smokers smoked sporadically, consuming less than a packet of cigaretes a week. This percentage of smokers in the population interviewed, despite being lower than that observed in the general Brazilian population (14.7%),^38^ is still significant, especially considering the level of physicians’ awareness about the risks associated with this habit.

A weakness of this study is not considering the prevalence of use of anxiolytic agents, antidepressants, analgesics, and medications used to improve sleep and psychosomatic symptoms, a topic that may be investigated in future studies, especially because physicians are a professional category with a very specific profile,^26^ susceptible to growing rates of burnout and depression.^10^

Another aspect that may represent a fall short of expectations, but provide subsidies for future studies, was the fact that we collected data up to the beginning of the COVID-19 pandemic. According to the Juiz de Fora City Hall, more than 820 deaths and 20 thousand cases were identified in the city up to March 2021.^39^ Therefore, new research is proposed afer the pandemic in order to assess possible changes from the perspective of this group of professionals in Juiz de Fora.

## CONCLUSIONS

The participating physicians in the had a good quality of life in all domains, which was influenced by sociodemographic characteristics such as sex, age, and time of professional experience. The highest score was observed in the physical health domain, followed by psychological, social relationships, and environment domains, in a descending order.

Results indicate that male sex, professional experience greater than 10 years, and age above 39 years are factors associated with beter quality. Conversely, previous illness and practice of presenteeism were associated with lower scores.

Most respondents reported having already fell ill, and a smaller portion reported taking sickness absence, whereas the levels of presenteeism were remarkably high, probably motivated by appreciation and awards to longer working hours and by the inability to remaining financially stable during sickness absence, considering that slightly more than a half of participants are covered by a labor insurance.

The most prevalent diseases during physician’s profession were those related to the respiratory system, infectious or parasitic diseases, and those related to the circulatory system, with a relevant number of mental and behavioral diseases.

This study is expected to contribute with the assessment of quality of life, illness, and factors related to this process, improving understanding about the implications of illness and presenteeism on physicians’ quality of life.
